# Health-Related Quality of Life following Total Thyroidectomy and Lobectomy for Differentiated Thyroid Carcinoma: A Systematic Review

**DOI:** 10.3390/curroncol29070350

**Published:** 2022-06-21

**Authors:** Vivianne Landry, Elizabeth Siciliani, Melissa Henry, Richard J. Payne

**Affiliations:** 1 Faculty of Medicine, University of Montreal, Montreal, QC H3T 1J4, Canada; vivianne.landry@mcgill.ca; 2 School of Communication Sciences and Disorders, McGill University, Montreal, QC H3A 0G4, Canada; 3 Faculty of Medicine, McGill University, Montreal, QC H3A 0G4, Canada; elizabeth.siciliani@mcgill.ca; 4 Gerald Bronfman Department of Oncology, McGill University, Montreal, QC H3A 0G4, Canada; melissa.henry@mcgill.ca; 5 Department of Otolaryngology-Head and Neck Surgery, McGill University Health Center, Montreal, QC H4A 3J1, Canada; 6 Lady-Davis Institute for Medical Research, Jewish General Hospital, Montreal, QC H3T 1E2, Canada; 7 Segal Cancer Centre, Jewish General Hospital, Montreal, QC H3T 1E2, Canada; 8 Department of Otolaryngology-Head and Neck Surgery, Jewish General Hospital, Montreal, QC H3T 1E2, Canada

**Keywords:** thyroid neoplasms, differentiated thyroid cancer, quality of life, thyroidectomy, lobectomy

## Abstract

Health-related quality of life (HrQoL) is a major concern for patients with differentiated thyroid carcinoma (DTC). We aimed to systematically review the literature comparing HrQol following total thyroidectomy (TT) and hemithyroidectomy (HT) in DTC patients. A systematic review of publications indexed in Medline, Embase, and EBM reviews—Cochrane Central Register of Controlled Trials, which evaluated HrQoL following thyroid surgery for DTC, was conducted. Of 2507 identified records, 25 fulfilled the inclusion criteria. Our results suggest that patients undergoing TT may suffer more impairment in physical and social HrQoL than patients undergoing HT. Psychological-related HrQoL and long-term global HrQoL are, however, equivalent in both groups, which highlights the multidimensional nature of HrQoL and the importance of a multitude of factors aside from treatment modalities and related morbidities, such as the experience of receiving a cancer diagnosis, the fear of cancer recurrence, and other psychosocial factors. Addressing postoperative HrQoL when discussing therapeutic options with patients is an integral part of patient-centered care and informed shared decision-making, and should be approached in a holistic manner, accounting for its physical, psychological, and social aspects. This review supplies evidence regarding HrQoL following thyroid surgery, which can be employed in such decisions.

## 1. Introduction

Over the last 50 years, the incidence of thyroid cancer has more than tripled in the United States, with mortality rates remaining largely stable over the last three decades [1,2]. The increased diagnosis of early thyroid malignancies has been accompanied by a rise in thyroid surgeries [3]. In the United States alone, it is estimated that over 150,000 thyroidectomies are performed annually [4]. Up to recently, the standard of care for patients presenting with a new diagnosis of thyroid cancer was total or near-total thyroidectomy (TT), with or without neck dissection, followed by radioactive iodine (RAI) treatment. This standard of care achieved an excellent prognosis: 5-year relative survival of 98.3% and recurrence rates of 3% in low-risk differentiated thyroid carcinoma (DTC), which accounts for the vast majority of new thyroid cancer diagnoses [5,6,7]. However, recognizing the indolent nature and excellent survival outcomes of most thyroid malignancies, recent guidelines have trended towards de-escalation of treatment for low-risk thyroid tumors. The British Thyroid Association (BTA) and the American Thyroid Association (ATA) both endorse subtotal or hemithyroidectomy (HT) for low-risk DTC of 1 to 4 cm, while TT is only strongly recommended for the initial management of higher-risk nodules [8,9]. These recommendations stem from large noncomparative national cohort studies finding similar outcomes between TT and HT regarding the prevention of recurrence and mortality [10,11,12,13]. It is well-known that more extensive surgeries (i.e., TT) are associated with an increased risk for intra- and postoperative complications (e.g., temporary or permanent recurrent laryngeal nerve palsy and hypoparathyroidism), which are expected to negatively impact patients’ HrqOL [14] For this reason, it has been hypothesized that HT, in addition to being a viable option from an oncologic standpoint, could be associated with a better postoperative HrQoL in low-risk DTC patients. However, the impact of thyroid surgery extent on patients’ health-related quality of life (HrQoL) remains poorly characterized in the literature. Despite DTC patients’ life expectancy being similar to that of the general population, their HrQoL may be impaired for up to 20 years following curative treatment [15,16,17,18,19]. Reasons for this long-term impairment in HrQol remain unclear but are likely multifactorial. Potential contributing factors include lingering physical symptoms (e.g., associated with calcium homeostasis issues, thyroid hormones imbalances, or voice impairment), the uncertainty regarding general health status, and concerns about the potential financial, psychological, and relational impacts of the DTC cancer diagnosis and its treatment [9] Given the high long-term survival rates and rising incidence of DTC, HrQoL remains a major concern for thyroid cancer patients and is an important factor to address during discussions surrounding therapeutic plans and long-term management. Recognizing that the appropriate extent of surgery in the management of DTC remains a recurrent subject of debate, we aim to systematically review the literature comparing TT and HT with regard to postoperative HrQoL with the goal of providing peer-reviewed information to clinicians that may be integrated into the shared treatment decision-making process with their patients.

## 2. Methods

### 2.1. Literature Search 

A systematic review of the literature was performed to identify relevant studies reporting on patients’ HrQoL following TT or HT. The PRISMA (Preferred Reporting Items for Systematic Reviews and Meta-Analyses) framework was used to guide the search and the reporting of the review [20]. The search strategy was created by our research team in collaboration with an experienced health-sciences librarian and validated for the three databases, namely Embase, Medline, and EBM reviews—Cochrane Central Register of Controlled Trials. This systematic review was registered on PROSPERO (International prospective register of systematic reviews; registration number: CRD42021255007). The search terms included in the search strategy are available in Supplementary Table S1. The search strategy is partially based on CADTH database’s search filters for health utilities and quality of life [21]. The search was conducted on 19 May 2021 for all three databases. References identified by hand-search, expert recommendations, or through reference lists of included studies were also considered.

### 2.2. Screening and Eligibility Assessment of Articles

References were assessed for eligibility according to the criteria outlined below (Section 2.3 and Section 2.4) in a two-step process. First, titles and abstracts of records obtained from the search strategy were screened for relevancy by two independent researchers. Then, all pertinent records were assessed by full-text reading against the eligibility criteria. Disagreements were resolved by consensus of all authors.

### 2.3. Inclusion Criteria

We included empirical studies conducted in human patients with a histopathologically confirmed diagnosis of well-differentiated thyroid cancer undergoing any thyroid surgery, with or without subsequent RAI ablation. For empirical studies using quantitative methodology, we included any studies comparing preoperative HrQoL with postoperative HrQoL, postoperative HrQoL with healthy controls, postoperative HrQoL with active surveillance, or postoperative HrQoL following TT vs. HT. For empirical studies using qualitative methodology, we included any studies providing information related to patients’ experiences with thyroid surgery, opinions related to the intervention, and life after the procedure such as satisfaction, preferences, and feelings. All included studies had to provide results according to surgery extent, and study groups had to be at least 95% homogenous in terms of surgical procedure performed (TT or HT). Due to limited resources for the translation of studies, only articles written in English or French were included.

### 2.4. Exclusion Criteria

Nonempirical studies were excluded (i.e., reviews, comments, letters, editorials, interviews). Studies including patients with medullary or anaplastic thyroid cancer were excluded. Studies reporting results only in the context of thyroid-hormone withdrawal or recombinant human TSH stimulation were also excluded.

### 2.5. Quality Assessment and Risk of Bias

Two researchers independently assessed the risk of bias using the Joanna Briggs Institute (JBI) critical-appraisal checklists, which allows for the assessment of quantitative and qualitative studies [22].

### 2.6. Definitions of TT vs. HT

For the purposes of this review, any surgical intervention restricted to one lobe of the thyroid, with or without isthmusectomy, was classified as a HT (lobectomy). Any bilateral resection, including total and near-total thyroidectomy, was classified as a total thyroidectomy. 

### 2.7. HrQoL Model

HrQoL is a multidimensional concept including physical and psychological symptoms, social wellbeing, and symptoms associated with illness or treatment [23]. Multiple HrQoL models have been developed over the years for use across a wide variety of health and disease conditions [23]. For the purposes of this review, we conceptualize HrQoL according to the Wilson and Cleary model, which emphasizes the importance of symptom status, general health perceptions, and functional status on overall HrQoL [24]. The data extracted from the included studies are thus classified according to three specific HrQoL components—physical, psychological, and social functioning—and global HrQoL. 

## 3. Results

### 3.1. Search-Strategy Results

**Figure 1:**
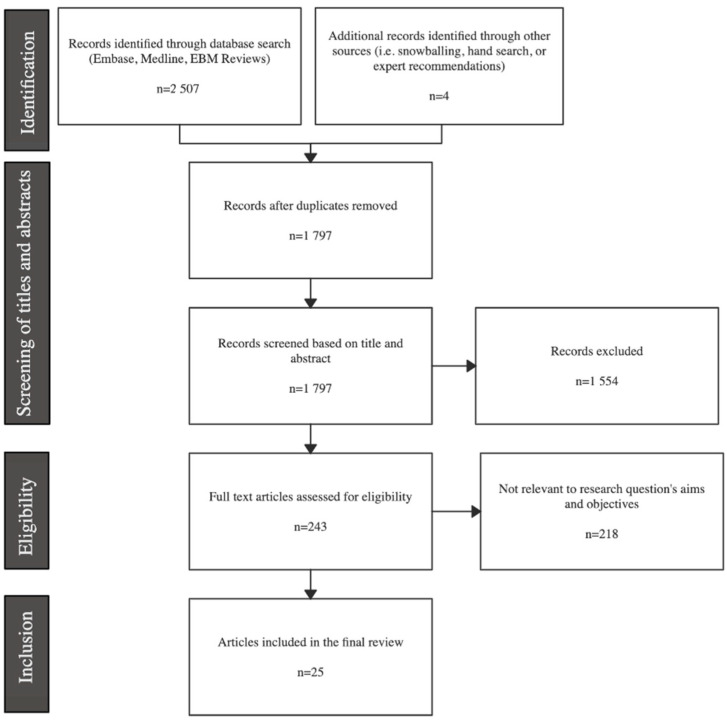
Prisma flow diagram.

The initial search resulted in a total of 2507 records that were imported into the Covidence systematic review software Version v2625 (Veritas Health Innovation, Melbourne, Australia) for screening and eligibility assessment (Figure 1). Four references were identified by hand-searched expert recommendations. After the removal of 714 duplicates and screening of all titles and abstracts, a total of 238 studies were assessed in full text, after which 25 articles were finally included in this review. 

### 3.2. Study Characteristics

Twenty-five studies were included from 13 different countries. Studies were mostly from China (*n* = 6), the United States (*n* = 5), the Netherlands (*n* = 3), and South Korea (*n* = 2). The remaining studies were from Canada (*n* = 1), Austria (*n* = 1), Sweden (*n* = 1), Finland (*n* = 1), Italy (*n* = 1), Greece (*n* = 1), Japan (*n* = 1), Australia (*n* = 1), and Egypt (*n* = 1). Twenty studies of quantitative design were included; two studies of qualitative and three studies of mixed methods were included. Nineteen studies were cross-sectional in nature, while six studies were transversal. Among transversal studies, four focused on early postoperative HrQoL (≤1 year following surgery), while two studies provided a long-term longitudinal assessment of HrQoL (≥2 years postoperatively and 4 years postoperatively). The postoperative time to HrQoL assessment varied greatly across studies, ranging from 1 week to 44 years. The majority of studies focused on TT patients (*n* = 14), while only one study focused exclusively on HT patients, and ten studies directly compared TT and HT patients. All studies evaluated DTC patients, with six studies including papillary thyroid microcarcinoma (PTMC) patients (Table 1).

### 3.3. HrQoL Instruments

In total, 23 different HrQoL instruments were used across included studies.

#### 3.3.1. SF-36 (or RAND-36)

The most commonly used instrument was the 36-item Short-Form Health Survey (SF-36 or RAND-36) employed in nine different studies. It is a general questionnaire comprised of 36 items measuring eight different scales [25,26]. Each of the items is scored and converted on a scale of 0 to 100 [26].

#### 3.3.2. SF-12

The 12-Item Short-Form Health Survey (SF-12) is an abbreviated version of its predecessor, the SF-36 [27]. It evaluates the same eight scales as the SF-36 and provides a score from 0 to 100 for each of the assessed items [27].

#### 3.3.3. SF-6D

The Short-Form Six Dimensions (SF-6D) is an 11-item general HrQoL instrument frequently used in economic evaluations [28]. It evaluates seven of the eight scales evaluated by its predecessor, the SF-36, by excluding the general health scale [28]. SF-6D utility values are scored from 0.3 to 1.0, where higher scores indicate better health [29].

#### 3.3.4. EQ5D-5L

The 5-Level EQ-5D (EQ-5D-5L) is a questionnaire comprised of 25 items and a visual analog scale (VAS) used in economic evaluations [30]. Each item is ranked on a scale of five levels and the VAS is graded on a scale ranging from 0 to 100. Scores are derived into an EQ-5D index value. In the United States, algorithm scores range from −0.11 to 1.0 [31,32].

#### 3.3.5. HUI2 and HUI 3

The Health Utilities Indexes 2 and 3 (HUI2 and HUI3) are 40-item health-status classification systems used in economic evaluations [33]. HUI2 scores range from −0.03 to 1.0, while HUI3 scores range from −0.36 to 1.0 [33].

#### 3.3.6. 15 D

The 15D instrument is a general HrQoL questionnaire assessing 15 different dimensions that can be used both as a profile and as a single index instrument [34]. 15D index scores range from 0 to 1, higher scores denoting better HrQoL [34,35].

#### 3.3.7. FoP

The Fear of Progression questionnaire (FoP) is a 43-item instrument used to assess anxiety and fear related to disease progression [36]. Items are scored on a scale of 0 to 4 [36].

#### 3.3.8. FoP-Q-SF

The Short Form of the Fear of Progression Questionnaire (FoP-Q-SF) is a 12-item version of its predecessor (FoP) [37]. Each item is scored on a scale from 1 to 5, with a resulting global score of 12 to 60 [37].

#### 3.3.9. ASC

The Assessment of Survivor’s Concerns (ASC) questionnaire includes six items evaluating cancer-related worries in cancer survivors [38]. Items are scored on a scale from 1 to 4, with higher scores reflecting greater cancer-related concerns [38].

#### 3.3.10. EORTC QLQ-C30

The European Organisation for Research and Treatment of Cancer’s Core Quality of Life Questionnaire (EORTC QLQ-C30) is a 30-item instrument designed to evaluate HrQoL in cancer patients [39]. All items are scored on a scale of 1 to 7, which is then transformed into a global score of 0 to 100 [39].

#### 3.3.11. EORTC QLQ-THY34

The EORTC Quality of Life Questionnaire’s supplementary module for thyroid cancer (EORTC QLQ-THY34) is a 34-item instrument meant to be employed in conjunction with the EORTC QLQ-C30 in thyroid cancer patients [40]. A score from 1 to 4 is attributed to each item, then transformed into a scale ranging from 0 to 100, with higher values indicating more impairment [40].

#### 3.3.12. THYCA-QoL

The THYCA-QoL is a 24-item questionnaire that was designed specifically to assess HrQoL in thyroid cancer survivors. Each item is ranked on a scale of 1–4 [41]. However, given that there is no scoring manual, different scoring methods are employed across studies, and minimum and maximum scores may vary.

#### 3.3.13. KT-QoL

The Korean Thyroid QoL questionnaire (KT-QOL) is the validated Korean translation of the QOL-Thyroid Scale, first developed by Dow et al. in the United States [42]. It is a 30-item instrument. Each item is scored on a scale of 0 to 10, with higher scores representing better HrQoL [43].

#### 3.3.14. ThyPRO

The ThyPRO questionnaire is an 84-item instrument designed to assess HrQoL in patients with benign thyroid disorders [44]. Scores range from 0 to 100, with high scores denoting more impairment [44].

#### 3.3.15. Billewicz Score 

The Billewicz Score is a clinical 14-item scale used to detect hypothyroidism. Its scores range from −47 to +67, with higher values denoting decreased thyroid function [45].

#### 3.3.16. MFI-20

The Multidimensional Fatigue Inventory (MFI-20) is a 20-item questionnaire measuring five different dimensions of fatigue [46]. Each subscale is scored from 4 to 20 and converted to a global fatigue score ranging from 20 to 100, with higher scores indicating more impairment [46].

#### 3.3.17. PSQI 

The Pittsburgh Sleep Quality Index (PSQI) is a 19-item instrument evaluating seven different components of sleep [47]. Global PSQI scores range from 0 to 21, where lower scores denote better sleep quality [47].

#### 3.3.18. HADS 

The Hospital Anxiety and Depression Scale (HADS) is a 14-item questionnaire, with seven items assessing depression and seven items assessing anxiety [48]. Each item is scored on a scale of 0 to 3, with a maximum score of 21 for each component [48].

#### 3.3.19. SDQ

The somatoform disorders questionnaire (SDW) assesses for the presence of somatoform disorders, as described in the third edition of the Diagnostic and Statistical Manual of Mental Disorders [18]. Scores for women range from 0 to 51, and for men, from 0 to 55 [18].

#### 3.3.20. State-Trait Anxiety Inventory

The State-Trait Anxiety Inventory (STAI) is a 40-item questionnaire used to assess an anxiety state at a given time in relation to basal anxiety levels [49]. Each item is scored on a scale of 1 to 4, which translates into a minimum global STAI score of 20 and a maximum score of 80, with higher levels reflecting greater anxiety [49].

#### 3.3.21. VHI

The Voice Handicap Index (VHI) is a 30-item instrument designed to assess the psychosocial impact of voice disorders. Scores range from 0 to 120, with higher scores denoting a greater handicap [50].

#### 3.3.22. VAS

A visual analog scale (VAS) was used by Teliti et al. to assess complaints related to medical therapy [51]. Scores ranged from 0 (no complaints) to 10 (worse degree of complaints) [51].

### 3.4. Quality Assessment

The JBI critical appraisal checklists were used to perform the quality assessment of included studies (Table 2, Table 3 and Table 4) [22]. Most studies lacked validity in outcome measurement and did not assess test-retest reliability. The overall quality of included studies was relatively homogenous according to study type and ranged from intermediate to high.

### 3.5. Physical HrQoL Component

In this review, we identified 20 studies reporting on physical HrQoL following TT or HT [18,34,51,52,53,54,55,56,57,58,59,60,61,62,64,65,67,68,70,73] (Table 5). Eight studies compared postoperative TT HrQoL with community reference values using seven different QoL instruments (i.e., SF-36, MFI-20, ThyPRO, PSQI, Billewicz Score, visual analog scale, and 15D) [18,34,51,53,55,58,61,68]. Among these studies, six found a significant decrease in HrQoL for at least one of the following items: bodily pain (*n* = 1), vitality (*n* = 2), physical functioning (*n* = 3), speech (*n* = 1), general or physical fatigue (*n* = 1), and sleep (*n* = 1) [18,34,53,55,58,61]. Interestingly, two of these studies also found a significant decrease in bodily pain (*n* = 1) or discomfort and symptoms (*n* = 1) following TT compared with the reference population [34,58]. Teliti et al. compared patients having undergone TT for DTC and for benign disease and found a significant impairment in the DTC group for goitre symptoms (*n* = 1), cognitive complaints (*n* = 1), and sleep efficiency (*n* = 1) [51]. One study found no significant differences between the TT group and the general population for physical-related HrQoL [68]. No study compared postoperative HT HrQoL with the general population’s HrQoL.

Three studies compared patients’ preoperative and postoperative physical-related HrQoL following TT [68,70,73]. One study found a significant increase in cognitive problems following TT, which were stable from two years following surgery up to four years following surgery [68]. Another study found a transitory impairment in voice quality following surgery, which decreased with time until there was, on average, no more vocal impairment at one year following surgery [70]. The last study found no significant difference between preoperative and postoperative HrQoL for the physical component [73]. No studies compared preoperative and postoperative HrQoL for HT patients.

Three studies assessed postoperative physical-related HrQoL in comparison with patients undergoing active surveillance (AS) [54,60,67]. All three studies found a significant impairment in physical-related HrQoL for patients undergoing HT versus patients undergoing AS for at least one of the following items: global physical subscale (*n* = 1), voice symptoms (*n* = 1), trouble with concentration (*n* = 2), neuromuscular symptoms (*n* = 1), throat or mouth symptoms (*n* = 2), feeling chilly (*n* = 1), or problems with scar (*n* = 2) [54,60,67]. For the two studies comparing postoperative TT HrQoL with AS, significant impairments were found in the surgery group for global physical subscale (*n* = 1), weight gain (*n* = 1), problems with scar (*n* = 1), and vocal symptoms (*n* = 1) [60,67]. One study found less impairment of libido in the HT group compared with the AS group [54].

Finally, nine studies directly compared physical-related HrQoL for patients undergoing TT versus HT using six different questionnaires (EORTC-QLQ-C30, EORTC-QLQ-THY34, SF-36, THYCA-QoL, FoP-Q-SF, and KT-QoL) [52,56,57,59,60,62,64,65,67]. Four of these studies found decreased physical-related HrQoL following TT compared with HT for at least one of the following items: global physical subscale (*n* = 1), voice symptoms (*n* = 1), sensory symptoms (*n* = 1), neuromuscular symptoms (*n* = 1), vitality (*n* = 1), and problems with scar (*n* = 1) [57,59,64,67]. One study found a transitory impairment in physical function, fatigue, pain, neuromuscular symptoms, vocal symptoms, libido, feeling chilly, or tingling symptoms in TT patients compared with HT patients between one and three months following surgery, although this difference was no longer significant at six months postoperatively. [65] Three studies found no significant differences between groups [56,60,62]. Two studies reported improved physical-related HrQoL in the TT group compared with the HT group for libido (*n* = 1) and cognitive functioning (*n* = 1) [52,57].

### 3.6. Psychological HrQoL Component

In this review, 19 studies reporting on psychological-related HrQoL were identified [18,34,51,52,53,54,55,56,57,58,59,60,61,63,64,65,67,68,73]. Eight of these studies assessed postoperative TT HrQoL in comparison with community reference values using six different instruments (SF-36, MFI-20, HADS, SDQ, Thy-PRO, 15D) [18,34,51,53,55,58,61,68]. Among these studies, five reported a significant decrease in postoperative psychological-related HrQoL compared with the general population for at least one of the following items: mental health component (*n* = 2), mental fatigue (*n* = 2), reduced motivation (*n* = 1), distress (*n* = 1), anxiety (*n* = 1), depression (*n* = 1), somatization (*n* = 1) [18,34,53,58,61]. Three studies reported no significant difference between the DTC TT group and the general population or patients having undergone TT for benign disease [51,55,68]. No studies compared postoperative HT psychological-related HrQoL with the general population. 

Only two studies compared preoperative and postoperative psychological-related HrQoL [68,73]. These studies focused solely on TT patients. One study found a significant increase in mental fatigue 2 years following TT, although anxiety and depression were decreased, and general mental health was improved 4 years following surgery [68]. The second study observed an improvement in mental health following surgery that was statistically significant one year postoperatively [73].

Three studies compared psychological-related HrQoL for patients undergoing surgery versus AS [54,60,67]. Two of these studies found no significant difference for psychological-related HrQoL between patients undergoing HT and patients undergoing AS, and one study described a significant decrease in mental health six months postoperatively that was no longer significant one year postoperatively [54,60,67]. The only study comparing TT patients with AS patients found a significant decrease in mental health from 6 months to 1.5 years postoperatively that was no longer significant after 2 years following surgery [67].

Finally, ten studies directly compared psychological-related HrQoL in TT and HT patients [52,56,57,59,60,62,63,64,65,67]. Four of these studies found no significant difference between groups for psychological-related HrQoL [57,60,64,67]. Four studies found a significant decrease in psychological-related HrQoL for the following items: psychological symptoms (*n* = 1), emotional functioning (*n* = 1), state anxiety (*n* = 1), and mental health (*n* = 1) for patients undergoing TT versus HT [56,59,62,63]. One study observed significantly more anxiety, depression, emotional functioning impairment, and psychological symptoms at one month postoperatively in TT patients versus HT patients [65]. However, this difference was no longer significant at three months postoperatively, with the exception of anxiety, which was no longer significant from six months following surgery onwards. Another study found improved psychological-related HrQoL in patients undergoing TT versus HT and reported a decrease in fear, worry (about cancer, recurrence, health, and overall worry), and alteration of body image [52].

### 3.7. Social HrQoL Component

In this review, 16 studies reporting on social functioning through 10 different instruments (SF-12, SF-36, MFI-20, ThyPRO, FoP-Q-SF, FoP, EORTC QLQ-C30, EORTC QLQ-THY34, KT-QoL, 15D) were identified [18,34,51,52,53,54,55,56,57,58,59,61,62,65,67,68]. Eight studies compared the social-related HrQoL of TT patients with the general population [18,34,51,53,55,58,61,68]. Five of these studies found significant impairment in social-related HrQoL in TT patients compared with the general population for at least one of the following items: social functioning and interactions (*n* = 4), role limitations due to emotional or physical health (*n* = 5), and reduced activity (*n* = 1) [18,51,53,55,58,61]. Two studies found no significant difference between TT patients and the general population for social functioning [34,68]. Teliti et al. compared patients having undergone TT for DTC versus benign disease and reported a greater impairment in the sexual life of DTC patients [51]. No studies assessed the social-related HrQoL of HT patients in comparison with healthy controls. 

One study compared patients’ preoperative and postoperative social-related HrQoL following TT, while no study evaluated this aspect in HT patients [68]. This study reported significantly less social impairment and impaired daily life at four years postoperatively, compared with preoperative time points [68].

Two studies compared the social-related HrQoL of patients undergoing surgery versus AS and found a significant decrease in the social subscale (*n* = 4), social functioning (*n* = 1), and role limitations due to physical or emotional health (*n* = 1) [54,67]. Of note, in one study, this impairment in social functioning was no longer statistically significant after two years following TT, although it was still significant at this time point for patients who underwent HT [67].

Finally, seven studies directly compared social-related HrQoL in TT and HT patients [52,56,57,59,62,65,67]. Three studies found decreased social-related HrQoL in TT patients compared with HT patients for the following items: social functioning (*n* = 2) and role limitations due to physical (*n* = 3) or emotional health (*n* = 2) [56,57,59]. One study found transitory impairment in the social HrQoL subscale for patients undergoing TT versus HT, a difference that subsided one year and a half postoperatively [67]. Similarly, another study observed a temporary impairment in social functioning, role functioning, and finances in patients undergoing TT versus HT between one and three months postoperatively, although this difference was no longer significative at later time points [65]. One study found improved social functioning, social support, and decreased impact on job for patients who underwent TT in comparison with patients who underwent HT [52]. One study observed no difference between groups [62].

### 3.8. Global HrQoL Component

Finally, 16 studies evaluated the global postoperative HrQoL in DTC patients using ten different instruments (SF-12, SF-36, SF-6D, EQ5D-5L, HUI2, HUI3, EORTC QLC-C30, ThyPRO, 15D, and KT-QoL) [18,34,52,53,54,55,56,57,58,59,62,65,66,67,68,73]. Six of these studies compared postoperative TT global HrQoL with the general population [18,34,53,55,58,68]. Among these, two found a decreased global HrQoL compared with community reference values [18,58]. One study including long-term DTC survivors having undergone TT (on average 5.5 years prior) found that TT patients had improved global HrQoL compared with the general population [53]. The three remaining studies found no significant difference between groups [34,55,68]. No studies compared HT patients’ HrQoL with community reference values.

Three studies compared preoperative versus postoperative global HrQoL following TT [66,68,73]. One of these studies observed no significant difference between groups, while one of them detected a significant improvement in overall HrQoL at one year postoperatively, and one of them observed improved QoL at 2 to 4 weeks postoperatively [66,68,73].

Two studies assessed patients’ global HrQoL following surgery or during AS [54,67]. One study found no significant difference between groups, while the other observed decreased HrQoL for patients undergoing HT at all time points, and decreased HrQoL for patients undergoing TT up to two years postoperatively [54,67].

Seven studies directly compared HT and TT patients in terms of global HrQoL [52,56,57,59,62,65,67]. Four of these studies observed no difference between groups [52,56,57,62]. One study found decreased general health in TT patients [59] The remaining two studies observed transitory impairments in global HrQoL in TT patients compared to HT patients, which subsided with time (6 months and 1 year postoperatively, respectively) [67].

### 3.9. Perspectives from Qualitative and Mixed Methods Studies

Qualitative studies are valuable in HrQoL research, as they allow for the consideration of individuals’ perceptions and interpretations; the understanding of their experiences of wellbeing; and the discovery of new issues related to HrQoL [74].

In this review, two different studies of qualitative and three studies of mixed methodology were identified, among which four focused on TT patients, and one focused on both TT and HT patients [69,70,71,72,73]. (Table 6). Three of these studies were based on semistructured interviews, one was based on structured phone interviews, and one was based on focus groups [69,70,71,72,73]. The types of analyses used ranged from grounded theory analysis (*n* = 2) to content analysis (*n* = 2) and thematic analysis (*n* = 1) [69,70,71,72,73].

Included qualitative studies identified themes related to physical symptoms (*n* = 4), psychological symptoms (*n* = 4), social functioning (*n* = 1), regret or dissatisfaction with treatment (*n* = 2), satisfaction with care provided (*n* = 1), and adaptative strategies (*n* = 2) [69,70,71,72,73].

Physical symptoms such as numbness and tingling, impaired communication, xerostomia, xeropthalmia, and dysgeusia were frequently reported (18–57%) following DTC treatment with TT [70,72,73]. Vocal symptoms and RAI treatment-related symptoms were perceived as deleterious to TT patients’ HrQoL, while hypoparathyroidism symptoms such as numbness and tingling did not have a substantial negative impact on patients’ overall HrQoL when adequately managed [70,72,73]. The study comparing TT and HT patients found that TT patients more often reported issues with overall physical health, medication, voice, fatigue, throat or neck discomfort, and other physical symptoms, while HT patients more commonly reported weight concerns and autonomic symptoms [71]. The authors concluded that HT, rather than TT, may lead to better HrQoL outcomes in selected DTC patients [71].

Psychological symptoms following TT such as distress, anxiety, and fear were described in three studies, although the incidence of such symptoms was not reported [69,72,73]. These symptoms were often reported in association with regrets or dissatisfaction with treatment or the healthcare system in general [69,72]. The study comparing TT and HT patients found that HT patients reported more psychological symptoms and emotional distress than TT patients, who in return reported more mood issues, anxiety, depression, and re-evaluation of life [71].

Themes relevant to social-related HrQoL were only described in two studies as “feeling supported” (*n* = 1) or “impact on work” (*n* = 1), which was more frequently reported by HT patients than TT patients [71,73].

Overall, the scarcity of qualitative studies focusing on HT patients does not allow a definite conclusion to be drawn regarding a difference between HT and TT patients’ HrQol. Nonetheless, it appears clear that DTC patients frequently struggle with both physical and psychological symptoms following surgery. Qualitative studies also emphasize the importance of an adequate therapeutic alliance when treating DTC patients. Relationships with the healthcare system could either be perceived as a source of anxiety or a source of support and strength by TT patients.

## 4. Discussion

### 4.1. Physical-Related HrQoL

Physical health and symptom status are often seen as the cornerstone of HrQoL. In thyroid disease, it has long been thought that symptoms related to thyroid dysfunction and treatment-related morbidity were the main contributing factors to postoperative impairment in HrQoL. It has previously been hypothesized that a lesser extent of surgery (i.e., HT) may be associated with better HrQoL due to the lower risk of surgical and post-operative complications, thus leading to better postoperative physical health [15,16,17,18]. Although TT may be associated with lower local recurrence rates, the risks of parathyroid injury and laryngeal nerve injury, which can potentially greatly impact HrQoL, are doubled [75,76,77].

We found that evidence regarding HrQoL following HT versus TT remains mitigated, with a tendency towards worse physical-related HrQoL in patients undergoing TT, which may be explained by the higher complication rates observed in the latter [75,76,77]. Given the relatively low incidence of complications reported in the literature, the small sample sizes of included studies may limit the detection of complication-related differences in HrQol [78]. The paucity of studies directly comparing HT and TT further limits the conclusions that can be drawn on this subject. More studies have focused on postoperative HrQoL in TT patients, and highlight the fact that HrQoL may be decreased in this patient population compared with the general population. Additionally, current evidence suggests that there may be a mild impairment in HrQoL for patients undergoing thyroid surgery (no matter the extent), compared with patients undergoing AS, although there is a small number of related publications (*n* = 3). While it has frequently been assumed that the decrease in HrQoL of patients with DTC was mostly attributed to treatment modalities and their associated complications, current evidence remains unclear on this matter. There is currently insufficient evidence to conclude that DTC patients’ postoperative HrQoL is worse than their preoperative HrQoL, which calls into question other possible contributive factors, such as the experience of receiving a cancer diagnosis, the fear of recurrence, and other psychosocial factors.

### 4.2. Psychological-Related HrQoL

The psychological impacts of cancer diagnoses are significant clinical issues to consider [79,80,81]. It has been argued that the distress related to cancer diagnosis and fear of recurrence may have just as much of an impact on HrQoL as physical symptoms [79,80]. It is known that DTC survivors experience higher levels of anxiety and depression than the general population and have similar levels of HrQoL than other cancer patients with worse prognoses [17,19,82]. So far, the psychological aspect of HrQoL in DTC patients has been poorly studied, with the majority of existing research focusing on the physical aspects of the disease and related treatments [81].

Current evidence seems to indicate that there are no significant differences in psychological-related HrQoL for patients undergoing HT versus TT, although the latter may be associated with less fear of recurrence or cancer-related worry. This relationship between fear of recurrence and surgery extent has seldom been explored in DTC but is extensively described in breast cancer by the trade-off hypothesis, i.e., the founded or unfounded rationale that an organ can be sacrificed or “traded” through radical surgery in exchange for a lower risk of recurrence [83]. Current evidence shows that psychological-related HrQoL is likely impaired in TT patients compared with the general population. This is consistent with our findings regarding physical-related HrQoL and previous research highlighting the fact that despite the excellent prognosis of the disease, HrQoL may be impaired in patients with thyroid neoplasms compared to the general population for up to 20 years following cancer treatment [15,16,17,18,19]. There seems to be no difference in psychological-related HrQoL for patients undergoing surgery versus active surveillance, which may suggest that the previously described impairment in psychological-related HrQoL may be strongly associated with the diagnosis of cancer rather than only the necessity for treatments or medical appointments. The fact that psychological-related HrQoL did not decrease but increased in some studies following surgery compared with preoperative time points further supports this hypothesis. The paucity of studies on this subject, however, limits the conclusions that can be drawn.

### 4.3. Social-Related HrQoL

Directly related to the physical and psychological components of HrQoL is the social functioning component; that is, the ability to fulfill social roles and maintain social relationships, interactions, and societal integration [84]. Adequate physical and psychological statuses indeed allow for healthy social functioning, which thus perhaps represents a more outcome-based component of HrQoL. Social functioning and integration may then in turn directly affect psychological and physical health. Thyroid cancer has long been thought of as a “good cancer” with limited impacts on social well-being [85,86]. The potential long-term impacts of DTC on social health are increasingly recognized as relevant clinical issues [87]. Current research indicates that social-related HrQoL may be decreased in patients undergoing TT versus HT, although the scarcity of related studies limits the strength of this conclusion. This finding is consistent with—and may also be explained by—the greater impairment in physical-related HrQoL that was observed in TT patients compared with HT patients. More studies have compared postoperative TT social-related HrQoL with community reference values and indicate that social wellbeing may be decreased in this patient population compared with the general population. There is currently insufficient evidence to conclude whether thyroid surgery affects social-related HrQoL in comparison with preoperative values or active surveillance. Social well-being remains understudied in DTC patients and more studies are needed in order to comprehend the true impact of thyroid disease and treatment on social-related HrQoL.

### 4.4. Global HrQoL

Current evidence suggests that there is no difference in long-term HrQoL between patients undergoing HT versus TT, although a greater impairment may be observed in TT patients in the first year following surgery. Current research is nuanced concerning a potential decrease in global HrQoL in TT patients compared with the general population. If this difference does exist, it may be partly due to the diagnosis of cancer, rather than only treatments and associated complications. This hypothesis is supported by the fact that no differences are observed between preoperative and postoperative global HrQoL in patients undergoing TT. Additionally, there is currently insufficient evidence to conclude to the existence of a difference in global HrQoL for patients undergoing surgery versus AS.

### 4.5. Clinical Implications

Current research highlights that, whether patients undergo TT or HT, the decision of undergoing thyroid surgery has far-reaching consequences that can affect long-term HrQoL. However, there has been limited research focused on developing interventions aimed at improving HrQoL in DTC patients. Nuria Javaloyes et al. presented a psycho-oncological intervention based on counseling, which successfully decreased anxiety and depression and increased psychological general well-being in DTC patients [88]. Wu et al. described a psychological and behavioral intervention that resulted in improvements in functional capacities, global QoL, and depression and anxiety symptoms at 1-year follow-up for DTC patients treated with surgery and RAI [89]. Henry et al. found that an interdisciplinary-team-based care approach including a nurse navigator improved overall well-being and improved levels of physical and practical concerns in patients treated for thyroid cancer [90]. More research is needed to address ways in which DTC patients’ postoperative HrQoL can be improved, including the use of preoperative tools to screen for distress and identify at-risk patients [91]. Addressing postoperative HrQoL when discussing therapeutic options with patients is an integral part of patient-centered care and informed shared decision-making, and should be approached in a holistic manner, accounting for its physical, psychological, social, and global aspects.

### 4.6. Limitations

Several limitations limit the conclusions that can be drawn from included studies. First, the important heterogeneity of included studies, both in terms of QoL instruments used, and postoperative times of assessment limited comparison across studies and did not allow for a meta-analysis to be performed. Second, the vast majority of included articles were cross-sectional, which limits the establishment of causal relationships. Third, there was a paucity of studies directly comparing HT and TT patients’ HrQoL or focusing on HT patients’ HrQoL in general. Fourth, the scope of our review did not allow us to assess the impact of thyroid surgery on the HrQoL of patients who are ultimately found to have benign diseases. These patients are expected to experience an entirely different set of challenges postoperatively, whether it is regarding the fear of cancer recurrence, the necessity for medical appointments, RAI ablation or external beam radiation therapy, or attitudes towards potential postoperative complications. To date, there has been limited research on postoperative HrQoL of this group of patients, although they represent a significant proportion of patients who undergo thyroid surgery [51,92,93,94]. Fifth, it is well-known that RAI treatment can lead to significant and potentially long-lasting side effects, and thus be detrimental to DTC patients’ HrQoL [95]. Due to the high proportion of TT patients having also undergone RAI ablation in the included studies, it is difficult to distinguish which variations in HrQoL can be attributed to the surgical versus radioactive treatment. Lastly, the inherently subjective and culturally sensitive nature of HrQoL assessed through patient-reported questionnaires could have contributed to the high heterogeneity of results across included studies.

## 5. Conclusions

HrQoL is increasingly being recognized as a significant clinical issue in thyroid cancer. This study provides an up-to-date synthesis of HrQoL following thyroid surgery that can help clinicians better counsel their patients while allowing for a more personalized approach, congruent with patients’ preferences and priorities. Our findings highlight the multidimensional nature of HrQoL and the importance of a multitude of factors aside from treatment choice and related morbidities, such as the experience of receiving a cancer diagnosis, the fear of cancer recurrence, and other psychosocial factors. Qualitative research furthermore emphasizes therapeutic alliances and relationships with healthcare providers as decisive factors in DTC patients’ HrQoL. Addressing postoperative HrQoL when discussing therapeutic options with patients is an integral part of patient-centered care and informed shared decision making, and should be approached in a holistic manner, accounting for its physical, psychological, social, and global aspects. As thyroid cancers are detected in greater numbers and in younger patients, HrQoL is an issue that will continue to gain importance over the next few years. More comprehensive, longitudinal, homogenous studies, including larger sample sizes, are needed in order to fully elucidate the impacts of TT and HT on DTC patients’ HrQoL.

## Figures and Tables

**Table 1:** Characteristics of included studies.

Characteristics	Number of Studies
Methodology	
Quantitative	20
Qualitative	2
Mixed methods	3
Cross-sectional	19
Transversal	6
Surgery extent	
TT	14
HT	1
Both	10
Comparisons for physical-related HrQoL	
HT vs. TT	9
Postoperative vs. Preoperative	3
Postoperative vs. Healthy controls	8
Postoperative vs. AS	3
Comparisons for psychological-related HrQoL	
HT vs. TT	10
Postoperative vs. Preoperative	2
Postoperative vs. Healthy controls	8
Postoperative vs. AS	3
Comparisons for social-related HrQoL	
HT vs. TT	7
Postoperative vs. Preoperative	1
Postoperative vs. Healthy controls	8
Postoperative vs. AS	2
QoL instruments and scores (minimum-maximum)	
15D instrument (0–1)	1
ASC (1–4)	1
Billewicz Score (−47–67)	1
EORTC QLC-C30 (0–100)	3
EORTC QLQ-THY34 (0–100)	1
EQ5D-5L (−0.11–1.0)	1
FoP (0–4)	1
FoP-Q-SF (1–5)	2
HADS (0–21)	4
HUI2 (−0.03 to 1.0)	1
HUI3 (−0.36–1.0)	1
KT-QoL (0–10)	1
MFI-20 (20–100)	3
PSQI. (0–21)	1
SDQ (0–51 (f.), 0–55 (m.))	1
SF-6D (0.3–1.0)	1
SF-12 (0–100)	2
SF-36 (0–100)	9
STAI (20–80)	1
ThyPRO (0–100)	2
THYCA-QoL	6
VHI (0–120)	1
Visual analog scale (0–10)	1

**Table 2:** Quality assessment of cross-sectional studies.

	Q1	Q2	Q3	Q4	Q5	Q6	Q7	Q8
Bongers et al. (2019) [52]	Y	Y	Y	Y	Y	Y	N	Y
Crevanna et al. (2003) [53]	N	N	U	Y	N	N/a	N	Y
Hoftijzer et al. (2007) [18]	Y	N	Y	Y	N	N/a	N	Y
Jeon et al. (2019) [54]	Y	Y	Y	Y	Y	Y	N	Y
Karapanou et al. (2012) [55]	Y	Y	Y	Y	Y	N	N	Y
Lan et al. (2020) [56]	Y	Y	Y	Y	Y	Y	N	Y
Lan et al. (2021) [57]	Y	Y	Y	Y	Y	Y	N	Y
Li et al. (2020) [58]	Y	Y	Y	Y	Y	Y	N	Y
Mlees et al. (2022) [59]	Y	Y	Y	Y	Y	Y	N	Y
Nakamura et al. (2020) [60]	Y	Y	Y	Y	N	N/a	N	Y
Nies et al. (2017) [61]	Y	N	Y	Y	Y	N/a	N	Y
Pelttari et al. (2009) [34]	Y	N	Y	Y	Y	Y	N	Y
Teliti et al. (2021) [51]	Y	Y	Y	Y	Y	Y	N	Y
Van Gerwen et al.(2022) [62]	Y	Y	Y	Y	Y	N	N	Y
Yang et al.(2022) [63]	Y	Y	Y	Y	Y	Y	N	Y
Zhang et al. (2020) [64]	Y	N	Y	Y	N	N/a	N	Y

**Q1.** Inclusion criteria were clearly defined? **Q2.** Subjects and settings were described in detail? **Q3.** Exposure was measured in a valid and reliable way? **Q4.** Objective, standard criteria were used to measure the condition? **Q5.** Confounding factors were identified? **Q6.** Strategies to deal with confounders were stated? **Q7.** Outcomes were measured in a valid and reliable way? **Q8.** Appropriate statistical analysis was used?

**Table 3:** Quality assessment of cohort studies.

	Q1	Q2	Q3	Q4	Q5	Q6	Q7	Q8	Q9	Q10	Q11
Chen et al. (2021) [65]	Y	Y	Y	Y	Y	N/a	N	Y	Y	Y	Y
Lubitz et al. (2017) [66]	Y	Y	Y	N	N	N/a	Y	Y	N	Y	Y
Moon et al. (2021) [67]	Y	Y	Y	N	N	N/a	N	Y	N	Y	Y
Van Velsen et al. (2019) [68]	Y	Y	Y	Y	Y	N/a	N	Y	N	Y	Y

**Q1.** The two groups were similar and recruited from the same population? **Q2.** The exposures were measured similarly to assign people to both exposed and unexposed groups? **Q3.** The exposure was measured in a valid and reliable way? **Q4.** Confounding factors were identified? **Q5.** Strategies to deal with confounders were stated? **Q6.** The participants were free of the outcome at the start of the study? **Q7.** Outcomes were measured in a valid and reliable way? **Q8.** The follow-up time was sufficient for outcomes to occur? **Q9.** Follow-up was complete, and if not, reasons for loss to follow-up were explored? **Q10.** Strategies to address incomplete follow-up were utilized? **Q11.** Appropriate statistical analysis was used?

**Table 4:** Quality assessment of mixed-methods or qualitative studies.

	Q1	Q2	Q3	Q4	Q5	Q6	Q7	Q8	Q9	Q10
Hedman et al. (2017) [69]	U	Y	Y	Y	Y	N	N	U	Y	Y
Kletzien et al. (2018) [70]	U	Y	Y	U	U	N	N	U	Y	Y
Nickel et al. (2019) [71]	U	Y	Y	Y	Y	N	N	U	Y	Y
Diamond Rossi et al. (2020) [72]	U	Y	Y	Y	Y	N	N	U	Y	Y
Doubleday et al. (2020) [73]	U	Y	Y	N	N	N	N	U	Y	Y

**Q1.** There is a congruity between the stated philosophical perspective and the research methodology? **Q2.** There is a congruity between the research methodology and the research question or objectives? **Q3.** There is a congruity between the research methodology and the methods used to collect data? **Q4.** There is a congruity between the research methodology and the representation and analysis of data? **Q5.** There is a congruity between the research methodology and the interpretation of results? **Q6.** There is a statement locating the researcher culturally or theoretically? **Q7.** The influence of the researcher on the research, and vice-versa, is addressed? **Q8.** Participants and their voices were adequately represented? **Q9.** The research is ethical according to current criteria, and there is evidence of ethical approval by an appropriate body? **Q10.** The conclusions drawn in the research report flow from the analysis, or interpretation, of the data?

**Table 5:** HrQoL outcomes after thyroid surgery (quantitative studies).

Study and Country	Sample Size	Surgery Extent	Pathology Details	RAI (%)	Morbidity Rates (%)	Postop ^a^ Time of QoL Assessment	QoL Instrument	QoL Items	Changes in QoL Scores									
									TT vs. HT				Postop vs. Preop ^b^		Postop vs. Healthy Controls		Postop vs. AS	
**Physical health component**																		
Crevanna et al. (2003) [53] Austria	150 51 (subgroup)	TT	DTC	100	RLNi ^f^: 3.3 HypoPTH ^g^: 16.6	0–23 y. (mean 5.5 y.)	SF-36	Bodily pain Physical functioning Vitality	-				-		**<1 y.** +4.1 −1.7 **−10.3**	**0–23 y.** **+5.4** −1.2 **−4.7**	-	
Hoftijzer et al. (2007) [18] The Netherlands	153	TT	DTC	100	N/a	0.3–41.8 y.	SF-36 MFI-20	Bodily pain Physical functioning General fatigue Physical fatigue	-				-		−2.43 **−4.07** **+2.43** **+2.35**		-	
Karapanou et al. (2012) [55] Greece	60	TT	PTC ^c^	100	N/a	2–6 m.	SF-36	Bodily pain Physical functioning Vitality	-				-		−0.09 **−6.88** −6.20		-	
Li et al. (2020) [58] China	174	TT	DTC	100	N/a	1 y.	SF-36	Bodily pain Physical functioning Vitality	-				-		**−7.4** −0.3 **−8.7**		-	
Nies et al. (2017) [61] The Netherlands	67	TT	DTC	97	Permanent hypoPTH: 25.4 RLNi: 14.9	5–44.7 y. (mean 17.8 y.)	SF-36 MFI-20	Bodily pain Physical functioning General fatigue Physical fatigue	-				-		−16 **−5** +1 +2		-	
Pelttari et al. (2009) [34] Finland	341	TT (98.8%)	94% PTC 6% FTC ^d^	84.5	RLNi: 1.4 Permanent hypoPTH: 1.7	5–19.5 y. (mean 12.4 y.)	15D instrument	Mobility Vision Hearing Breathing Sleeping Eating Speech Elimination Discomfort and symptoms Vitality Sexual activity	-				-		+0.010 +0.005 −0.013 −0.002 **−0.036** −0.003 **−0.017** +0.005 **+0.044** −0.011 −0.019		-	
Teliti et al. (2021) [51] Italy	119	TT (95.6%)	DTC	71	N/a	Mean 9.9 y.	PSQI ThyPRO Billewicz score VAS	Global PSQI Sleep quality Sleep latency Sleep duration Sleep efficiency Sleep disturbance Use of sleep medication Daytime dysfunction Goiter symptoms Hyperthyroid symptoms Hypothyroid symptoms Eye symptoms Tiredness Cognitive problems Cosmetic complaints Hypothyroid symptoms Complaints due to medical therapies	-				-		+0.985 +0.093 −0.049 +0.1799 **+0.394** +0.083 +0.013 +0.283 **+2.00** +0.78 +0.517 +1.134 −0.52 **+7.04** +1.227 = +0.6		-	
Van Velsen et al. (2019) [68] The Netherlands	185	TT	88% PTC 12% FTC	100	RLNi: 9.2 Transient hypoPTH: 20.0 Permanent hypoPTH: 16.8	2–4 y.	MFI-20 SF-36 ThyPRO	General fatigue Physical fatigue Physical functioning Vitality Tiredness Cognitive problems	-				**2 y.** +0.7 +0.2 −2.4 −2.7 +1.0 **+4.9**	**4 y.** +0.6 = −0.8 −2.3 +1.4 **+5.0**	**2 y.^☨^** +5.1 +4.8 -8.0 -13.5 +6.6 +0.3	**4 y.^☨^** +5.0 +4.6 -6.4 -13.1 +7.0 +0.4	-	
Doubleday et al. (2020) [73] USA	62	TT	DTC	N/a	Transient hypoPTH: 4.8	2 w. 6 w. 6 m. 1 y.	SF-12	Physical component	-				−8.07 −1.40 +0.65 +1.49		-		-	
Kletzien et al. (2018) [70] USA	42	TT	PTC	N/a	RLNi: 19.0	2 w. 6 w. 6 m. 1 y.	VHI	Voice impairment (total)	-				**+9.70** +8.70 +1.32 −1.42		-		-	
Chen et al. (2021) [65] China	427 365	HT TT	DTC	2.3 18.1	Transient hypoPTH: 6.6 RLNi: 0.4 Transient hoarseness: 21.1 Transient hypoPTH: 32.2 RLNi: 1.6 Transient hoarseness: 30.8	1 m.–1 y.	EORTC QLC-C30 THYCA-QoL	Physical functioning Cognitive functioning Fatigue Nausea/vomiting Pain Dyspnoea Sleep disturbances Appetite loss Constipation Diarrhea Neuromuscular Voice Concentration Sympathetic Throat/mouth Sensory Problems with scar Feeling chilly Tingling hands/feet Weight gain Headache Decreased libido	**1 m** **−2.1** 0.6 **3.2** −0.03 **2.7** −0.3 0.5 −0.03 −0.25 −0.5 **2.7** **8.2** 0.5 1.9 2.3 −1.7 3.0 **2.9** **2.4** 1.2 −1.6 **−3.9**	**3 m** **−2.7** −0.3 **2.97** 0.2 0.5 −0.4 −0.3 **1.57** −0.1 −0.4 **3.6** **3.8** 0.1 **2.4** 2.1 −0.5 2.2 0.6 **3.1** 2.95 −0.5 −0.9	**6 m** −0.1 1.98 −0.7 0.02 0.8 −1.1 −0.5 0.15 −0.4 0.05 1.1 0.1 −1.4 0.9 −0.45 −1.1 1.5 0.4 1.1 0.05 −1.8 **−2.3**	**1 y.** 0.2 1.8 −1.7 −0.2 0.05 1.0 −3.2 −1.1 −0.6 0.2 0.1 −0.9 −1.6 1.7 −0.2 −0.45 0.9 −0.9 −0.4 −2.5 −0.6 1.0	**-**		-		-	
Bongers et al. (2019) [52] Canada	59 211	HT TT	47.5% PTC 52.5% FVPTC ^e^ 33.6% PTC 65.4% FVPTC 0.9% FTC	0 43.6	Permanent hypoPTH: 0.0 Persistent RLNi: 0.0 Permanent hypoPTH: 6.2 Persistent RLNi: 0.0	0.9–12.7 y.	EORTC QLC-C30 EORTC QLQ-THY34	Physical functioning Cognitive functioning Fatigue Nausea/vomiting Pain Dyspnoea Sleep disturbances Appetite loss Constipation Diarrhea Fatigue Neck discomfort Voice concerns Hair problems Swallowing Dry mouth Temperature intolerance Restlessness Shoulder function Joint pain Tingling/numbness Cramps	+0.1 **+6.5** −4.2 +1.2 +2.0 +0.3 −8.4 −0.5 −0.6 +0.3 −5.0 −2.8 +0.4 +2.5 +1.5 +0.6 −6.2 −1.1 −1.7 +4.2 +0.2 +3.8				**-**		**-**		**-**	
Lan et al. (2021) [57] China	34 35	HT TT	PTMC	0	RLNi: 0.0 RLNi: 8.6	0-45 m.	SF-36 THYCA-QoL FoP-Q-SF	Bodily pain Physical functioning Vitality Neuromuscular Voice Concentration Sympathetic Throat/mouth Sensory Problems with scar Feeling chilly Tingling hands/feet Weight gain Headache Decreased libido Physical health	−9 = −5 +11 = = +16 = −8 **+33** = = +16 −17 **−33** =				-		-		-	
Lan et al. (2020) [56] China	18 16	HT TT	PTMC	N/a	RLNi: 5.9	Mean 20.29 m.	SF-36 THYCA-QoL FoP-Q-SF	Bodily pain Physical functioning Vitality Neuromuscular Voice Concentration Sympathetic Throat/mouth Sensory Problems with scar Feeling chilly Tingling hands/feet Weight gain Headache Decreased libido Physical health	−3.34 −2.39 −11.8 +4.09 −7.18 +5.79 +7.87 −2.78 +4.16 +9.49 −0.93 +4.63 +6.25 +3.47 −4.4 +0.28				-		-		-	
Mlees et al. (2022) [59] Egypt	42 40	HT TT	Minimally invasive FTC	N/a	Transient hypoPTH: 2.4 Transient hypoPTH: 12.5 Permanent hypoPTH: 2.5 Temporary RLNi: 7.5 Persistent RLNi: 2.5 Seroma: 7.5 Infection: 5.0	12 m.	SF-36	Bodily pain Physical functioning Vitality	−2.8 −1.8 **−5.3**				-		-		-	
Van Gerwen et al. (2021) [62] USA	34 24	HT TT	84.4% PTC 15.5% FTC	0	N/a	2–20 y.	EORTC QLC-C30	Physical functioning Cognitive functioning Fatigue Nausea/vomiting Pain Dyspnoea Insomnia Appetite loss Constipation Diarrhea	−6.8 3.6 3.6 1.1 7.6 9.1 9.6 −0.8 5.5 −2.9				-		-		-	
Zhang et al. (2020) [64] China	19 8	HT TT	PTMC	N/a	Persistent RLNi: 2.5 Permanent hypoPTH: 0.0 Persistent RLNi: 7.1 Permanent hypoPTH: 7.1	Median: 63.6 m.	THYCA-QoL	Neuromuscular Voice Concentration Sympathetic Throat/mouth Sensory Problems with scar Feeling chilly Tingling hands/feet Weight gain Headache Decreased libido	**=** **+0.72** = −0.13 −0.14 **+0.05** −0.32 −0.21 −0.11 −0.16 +0.14 =				**-**		**-**		**-**	
Moon et al. (2020) [67] South Korea	238 79 500	HT TT AS	PTMC	N/a	N/a	6 m. 1 y 1.5 y. ≥2. Y.	KT-QoL	Physical subscale	**−0.6** −0.4 −0.5 0.004								**HT** **−0.7** **−0.5** **−1.1** **−0.5**	**TT** **−1.3** **−0.9** **−1.5** −0.5
Nakamura et al. (2020) [60] Japan	17 32 298	HT TT AS	PTMC	N/a	Temporary RLNi: 6 Persistent RLNi: 0 Transient hypoPTH: 33 Permanent hypoPTH: 4 -	64–130 m. (mean: 84 m.)	THYCA-QoL	Neuromuscular Voice Concentration Sympathetic Throat/mouth Sensory Problems with scar Feeling chilly Tingling hands/feet Weight gain Headache Decreased libido	−11 −17 −33 +17 = = = = = +33 +33 =				-		-		**HT** +11 **+17** **+33** = = = **=** = = = = =	**TT** = **=** = +17 = = **=** = = **+33** +33 =
Jeon et al. (2019) [54] South Korea	148 43	HT AS	PTMC	0	Transient hypoPTH: 1.4 -	14.2–53.0 m.	SF-12 THYCA-QoL	Bodily pain Physical functioning Vitality Neuromuscular Voice Concentration Sympathetic Throat/mouth Sensory Problems with scar Feeling chilly Tingling hands/feet Weight gain Headache Decreased libido	-				-		-		−12.04 −2.05 −0.94 **+5.72** +2.95 **+5.03** +4.99 **+5.98** +1.18 **+9.12** **+4.87** +4.17 +5.08 +2.90 **−9.27**	
**Psychological health component**																		
Crevanna et al. (2003) [53] Austria	150 51 (sub-group)	TT	DTC	100	RLNi: 3.3 HypoPTH: 16.6	0–23 y. (mean: 5.5 y.)	SF-36	Mental health	-				-		**<1 y.** **−8.14**	**0–23 y.** −2.46	-	
Hoftijzer et al. (2007) [18] Netherlands	153	TT	DTC	100	N/a	0.3–41.8 y.	MFI-20 HADS SDQ	Reduced motivation Mental fatigue Anxiety Depression Anxiety + depression Somatization	-				-		**+1.38** **+1.61** **+1.48** **+0.75** **+2.23** **+4.27**			
Karapanou et al. (2012) [55] Greece	60	TT	PTC	100	N/a	2–6 m.	SF-36	Mental health	-				-		+2.46		-	
Li et al. (2020) [58] China	174	TT	DTC	100	N/a	1 y.	SF-36	Mental Health	-				-		**−10.2**		-	
Nies et al. (2017) [61] Netherlands	67	TT	DTC	97	Permanent hypoPTH: 25.4 RLNi: 14.9	5–44.7 y. (mean 17.8 y.)	SF-36 MFI-20 HADS	Mental Health Reduced motivation Mental fatigue Anxiety Depression	-				-		= = **+2** +1 =		-	
Pelttari et al. (2009) [34] Finland	341	TT (98.8%)	94.5% PTC 5.5% FTC	84.5	RLNi: 1.4 Permanent hypoPTH: 1.7	5–19.5 y. (mean 12.4 y.)	15D instrument	Mental function Depression Distress	-				-		+0.003 −0.014 **−0.023**		-	
Teliti et al. (2021) [51] Italy	119	TT (95.6%)	DTC	71	N/a	Mean 9.9 y.	ThyPRO ^g^	Anxiety Depressivity Emotional susceptibility	-				-		+0.407 +0.554 +0.38		-	
Van Velsen et al. (2019) [68] Netherlands	185	TT	88% PTC 12% FTC	100	RLNi: 9.2 Transient hypoPTH: 20.0 Permanent hypoPTH: 16.8	2-4 y.	MFI-20 SF-36 ThyPRO	Mental fatigue Mental health Anxiety Depressivity	-				**2 y.** **+0.7** +1.5 **−13.1** **−5.8**	**4 y.** +0.4 **+2.6** **−13.8** **−5.9**	**2 y.^☨^** +4.7 −8.6 +0.6 −2.9	**4 y.^☨^** +4.4 −7.5 −0.1 −3.0	-	
Doubleday et al. (2020) [73] USA	62	TT	DTC	N/a	Transient hypoPTH: 4.8	2 w. 6 w. 6 m. 1 y.	SF-12	Mental health component	-				+5.04 +3.58 +3.61 **+4.84**		-		-	
Chen et al. (2021) [65] China	427 365	HT TT	DTC	2.3 18.1	Transient hypoPTH: 6.6 RLNi: 0.4 Transient hoarseness: 21.1 Transient hypoPTH: 32.2 RLNi: 1.6 Transient hoarseness: 30.8	1 m.–1 y.	EORTC QLC-C30 THYCA-QoL HADS	Emotional function Psychological symptoms Anxiety Depression	**1 m.** **−3.2** **2.3** **0.6** **0.4**	**3 m.** −0.9 0.5 **0.4** 0.3	6 **m.** 1.0 −1.1 −0.05 −0.1	**1 y.** −0.6 −0.45 0.1 −0.15	-		-		**-**	
Bongers et al. (2019) [52] Canada	59 211	HT TT	47.5% PTC 52.5% FVPTC ^e^ 33.6% PTC 65.4% FVPTC 0.9% FTC	0 43.6	Permanent hypoPTH: 0.0 Persistent RLNi: 0.0 Permanent hypoPTH: 6.2 Persistent RLNi: 0.0	0.9–12.7 y.	EORTC QLC-C30 EORTC QLQ-THY34 ASC	Emotional functioning Body image altered Fear Worry Cancer worry Future test worry New cancer worry Recurrence worry General health worry Death worry Health worry Overall worry	+3.8 **−5.5** **−5.2** −7.9 **−0.7** −0.2 −0.2 **−0.3** −0.4 = **−0.3** **−1.1**				-		**-**		**-**	
Lan et al. (2021) [57] China	34 35	HT TT	PTMC	0	RLNi: 0.0 RLNi: 8.6	0–45 m.	SF-36 THYCA-QoL	Mental health Psychological	−10 +8				-		**-**		**-**	
Lan et al. (2020) [56] China	18 16	HT TT	PTMC	N/a	RLNi: 5.9	Mean 20.29 m.	SF-36 THYCA-QoL	Mental health Psychological	−2.58 **+12.8**				-		-		-	
Mlees et al. (2022) [59] Egypt	42 40	HT TT	Minimally invasive FTC	N/a	Transient hypoPTH: 2.4 Transient hypoPTH: 12.5 Permanent hypoPTH: 2.5 Temporary RLNi: 7.5 Persistent RLNi: 2.5 Seroma: 7.5 Infection: 5.0	12 m.	SF-36	Mental health	**−4.1**				-		-		-	
Van Gerwen et al. (2021) [62] USA	34 24	HT TT	84.4% PTC 15.5% FTC	0	N/a	2–20 y.	EORTC QLC-C30	Emotional functioning	**−5.4**				-		-		-	
Yanf et al. (2022) [63] China	86 263	HT TT	PTC	N/a	N/a	<1 w.–1 mo.	STAI	State anxiety	**1.39**				-		-		-	
Zhang et al. (2020) [64] China	19 8	HT TT	PTMC	N/a	Persistent RLNi: 2.5 Permanent hypoPTH: 0.0 Persistent RLNi: 7.1 Permanent hypoPTH: 7.1	Median: 63.6 m.	THYCA-QoL	Psychological problems	−0.22				**-**		**-**		**-**	
Moon et al. (2020) [67] South Korea	238 79 500	HT TT AS	PTMC	N/a	N/a	6 m. 1 y 1.5 y. ≥2 y.	KT-QoL	Mental health subscale	−0.4 −0.4 −0.7 0.11								**HT** **−0.4** −0.3 −0.5 −0.2	**TT** **−0.8** **−0.7** **−1.2** −0.1
Nakamura et al. (2020) [60] Japan	17 32 298	HT TT AS	PTMC	N/a	Temporary RLNi: 6 Persistent RLNi: 0 Transient hypoPTH: 33 Permanent hypoPTH: 4 -	64–130 m. (mean: 84 m.)	THYCA-QoL HADS	Psychological Anxiety Depression Total	= −1 = −1				-		-		**HT** +9 +2 +2 +3	**TT** +9 +1 +2 +2
Jeon et al. (2019) [54] South Korea	148 43	HT AS	PTMC	0	Transient hypoPTH: 1.4 -	14.2 m.–53.0 m.	SF-12 THYCA-QoL FoP	Mental health Psychological Affective reactions Coping with anxiety	-				-		-		−1.01 +2.57 −0.05 −0.11	
**Social functioning component**																		
Crevanna et al. (2003) [53] Austria	150 51 (sub-group)	TT	DTC	100	RLNi: 3.3 HypoPTH: 16.6	0–23 y. (mean 5.5 y.)	SF-36	Role limitations due to emotional health Role limitations due to physical health Social functioning and interactions	-				-		**<1 y.** **−30.2** **−18.3** **−9.19**	**0–23 y.** **−16.39** −10.92 −3.19	-	
Hoftijzer et al. (2007) [18] Netherlands	153	TT	DTC	100	N/a	0.3–41.8 y.	SF-36 MFI-20	Role limitations due to emotional health Role limitations due to physical health Social functioning and interactions Reduced activity	-				-		−2.71 **−8.03** **−6.97** **+1.61**		-	
Karapanou et al. (2012) [55] Greece	60	TT	PTC	100	N/a	2–6 m.	SF-36	Role limitations due to emotional health Role limitations due to physical health Social functioning and interactions	-				-		**−7.22** **−11.25** **−9.17**		-	
Li et al. (2020) [58] China	174	TT	DTC	100	N/a	1 y.	SF-36	Role limitations due to emotional health Role limitations due to physical health Social functioning and interactions	-				-		**−10.0** **−7.1** **−7.6**		-	
Nies et al. (2017) [61] Netherlands	67	TT	DTC	97	Permanent hypoPTH: 25.4 RLNi: 14.9	5–44.7 y (mean 17.8 y.)	SF-36 MFI-20	Role limitations due to emotional health Role limitations due to physical health Social functioning and interactions Reduced activity	-				-		= **=** −12 =		-	
Pelttari et al. (2009) [34] Finland	341	TT (98.8%)	94.5% PTC 5.5% FTC	84.5	RLNi: 1.4 Permanent hypoPTH: 1.7	5–19.5 y. (mean 12.4 y.)	15D instrument	Usual activities	-				-		+0.004		-	
Teliti et al. (2021) [51] Italy	119	TT (95.6%)	DTC	71	N/a	Mean 9.9 y.	ThyPRO ^g^	Social life impairment Daily life impairment Sex life impairment	-				-		+0.074 +1.199 **+0.825**		-	
Van Velsen et al. (2019) [68] Netherlands	185	TT	88% PTC 12% FTC	100	RLNi: 9.2 Transient hypoPTH: 20.0 Permanent hypoPTH: 16.8	2–4 y.	SF-36 ThyPRO	Social functioning Social life impairment Daily life impairment	-				**2 y.** +3.2 −0.9 −2.3	**4 y.** +3.6 **−1.5** **−3.0**	**2 y.^☨^** −16.8 N/a N/a	**4 y.^☨^** −16.4 N/a N/a	-	
Chen et al. (2021) [65] China	427 365	HT TT	DTC	2.3 18.1	Transient hypoPTH: 6.6 RLNi: 0.4 Transient hoarseness: 21.1 Transient hypoPTH: 32.2 RLNi: 1.6 Transient hoarseness:30.8	1 m.–1 y.	EORTC QLC-C30	Role function Social function Financial difficulties	**1 m.** −2.8 **−3.7** 2.3	**3 m.** **−2.96** **−2.9** **3.8**	6 **m.** 1.1 −0.9 2.5	**1 y.** 1.1 1.4 0.5	-		**-**		**-**	
Bongers et al. (2019) [52] Canada	59 211	HT TT	47.5% PTC 52.5% FVPTC ^e^ 33.6% PTC 65.4% FVPTC 0.9% FTC	0 43.6	Permanent hypoPTH: 0.0 Persistent RLNi: 0.0 Permanent hypoPTH: 6.2 Persistent RLNi: 0.0	0.9–12.7 y.	EORTC QLC-C30 EORTC QLQ-THY34	Role functioning Social functioning Financial difficulties Impact on job Social support	+2.6 **+5.8** −4.8 **−10.7** **+6.7**				-		**-**		**-**	
Lan et al. (2021) [57] China	34 35	HT TT	PTMC	0	RLNi: 0.0 RLNi: 8.6	0–45 m.	SF-36 FoP-Q-SF^i^	Role limitations due to emotional health Role limitations due to physical health Social functioning and interactions Social family	**−33** **−50** **−11** =				-		**-**		**-**	
Lan et al. (2020) [56] China	18 16	HT TT	PTMC	N/a	RLNi: 5.9	Mean 20.3 m.	SF-36 FoP-Q-SF	Role limitations due to emotional health Role limitations due to physical health Social functioning and interactions Social family	−22.4 **−31.8** **−10.3** +0.26				-		-		-	
Mlees et al. (2022) [59] Egypt	42 40	HT TT	Minimally invasive FTC	N/a	Transient hypoPTH: 2.4 Transient hypoPTH: 12.5 Permanent hypoPTH: 2.5 Temporary RLNi: 7.5 Persistent RLNi: 2.5 Seroma: 7.5 Infection: 5.0	12 m.	SF-36	Role limitations due to emotional health Role limitations due to physical health Social functioning and interactions	**−5.6** **−3.2** +1.9				-		-		-	
Van Gerwen et al. (2021) [62] USA	34 24	HT TT	84.4% PTC 15.5% FTC	0	N/a	2–20 y.	EORTC QLC-C30	Role functioning Social functioning Financial difficulties	−8.4 −13.1 11.6				-		-		-	
Moon et al. (2020) [67] South Korea	238 79 500	HT TT AS	PTMC	N/a	N/a	6 m. 1 y 1.5 y. ≥2 y.	KT-QoL	Social subscale	**−0.5** **−0.7** −0.3 0.3								**HT** **−0.7** **−0.4** **−1.0** **−0.7**	**TT** **−1.2** **−1.0** **−1.3** −0.4
Jeon et al. (2019) [54] South Korea	148 43	HT AS	PTMC	0	Transient hypoPTH: 1.4 -	14.2–53.0 m.	SF-12 FoP	Role limitations due to emotional health Role limitations due to physical health Social functioning Partnership/family Work Loss of autonomy	-				-		-		**−3.09** **−2.42** **−2.17** +0.03 +0.08 −0.14	
**Global HrQoL**																		
Crevanna et al. (2003) [53] Austria	150 51 (sub-group)	TT	DTC	100	RLNi: 3.3 HypoPTH: 16.6	0–23 y. (mean 5.5 y.)	SF-36	General health	-				-		**<1 y.** +0.5	**0–23 y.** **+4.4**	-	
Hoftijzer et al. (2007) [18] Netherlands	153	TT	DTC	100	N/a	0.3–41.8 y.	SF-36	General health Change in health	-				-		**−5.75** −2.62		-	
Karapanou et al. (2012) [55] Greece	60	TT	PTC	100	N/a	2–6 m.	SF-36	General health	-				-		−4.14		-	
Li et al. (2020) [58] China	174	TT	DTC	100	N/a	1 y.	SF-36	General health	-				-		**−11.7**		-	
Pelttari et al. (2009) [34] Finland	341	TT (98.8%)	95% PTC 5% FTC	84.5	RLNi: 1.4 Permanent hypoPTH: 1.7	5–19.5 y. (mean 12.4 y.)	15D instrument	Global score	-				-		−0.002		-	
Van Velsen et al. (2019) [68] Netherlands	185	TT	88% PTC 12% FTC	100	RLNi: 9.2 Transient hypoPTH: 20.0 Permanent hypoPTH: 16.8	2–4 y.	SF-36 ThyPRO	General health perception Composite score	-				**2 y.** −0.5 −1.7	**4 y.** −1.0 −2.5	**2 y.^☨^** −15.3 N/a	**4 y.^☨^** −15.8 N/a	**-**	
Doubleday et al. (2020) [73] USA	62	TT	DTC	N/a	Transient hypoPTH: 4.8	2 w. 6 w. 6 m. 1 y.	EORTC QLC-C30	Global scale	-				−3.63 +0.65 +8.38 **+11.6**		-		-	
Lubitz et al. (2017) [66] USA	95	TT (96%)	74% PTC 16% FVPTC	49	HypoPTH: 5.0 Hematoma: 1.0 RLNi: 3.3	2–4 w. 6–12 m.	EQ5D-5L SF-6D HUI2 HUI3	Global scores	-				**2–4 w.** 0.02 **0.04** 0.01 0.02	**6–12 m** −0.00 −0.01 0.01 0.02	**-**		-	
Chen et al. (2021) [65] China	427 365	HT TT	DTC	2.3 18.1	Transient hypoPTH: 6.6 RLNi: 0.4 Transient hoarseness: 21.1 Transient hypoPTH: 32.2 RLNi: 1.6 Transient hoarseness: 30.8	1 m.–1 y	EORTC QLC-C30	Global scale	**1 m.** −2.1	**3 m.** **−2.9**	6 **m.** −1.0	**1 y.** −0.04	-		-		-	
Bongers et al. (2019) [52] Canada	59 211	HT TT	47.5% PTC 52.5% FVPTC ^e^ 33.6% PTC 65.4% FVPTC 0.9% FTC	0 43.6	Permanent hypoPTH: 0.0 Persistent RLNi: 0.0 Permanent hypoPTH: 6.2 Persistent RLNi: 0.0	0.9–12.7 y.	EORTC QLC-C30	Global scale	+0.80				-		-		-	
Lan et al. (2021) [57] China	34 35	HT TT	PTMC	0	RLNi: 0.0 RLNi: 8.6	0–45 m.	SF-36	General health	−4.00				-		**-**		**-**	
Lan et al. (2020) [56] China	18 16	HT TT	PTMC	N/a	RLNi: 5.9	Mean 20.29 m.	SF-36	General health	−4.72				-		-		-	
Mlees et al. (2022) [59] Egypt	42 40	HT TT	Minimally invasive FTC	N/a	Transient hypoPTH: 2.4 Transient hypoPTH: 12.5 Permanent hypoPTH: 2.5 Temporary RLNi: 7.5 Persistent RLNi: 2.5 Seroma: 7.5 Infection: 5.0	12 m.	SF-36	General health	**−3.1**				-		-		-	
Van Gerwen et al. (2021) [62] USA	34 24	HT TT	84.4% PTC 15.5% FTC	0	N/a	2–20 y.	EORTC QLC-C30	Global health	−1.9				-		-		-	
Moon et al. (2020) [67] South Korea	238 79 500	HT TT AS	PTMC	N/a	N/a	6 m. 1 y 1.5 y. ≥2. y.	KT-QoL	Global subscale	**−0.5** **−0.4** −0.5 −0.02								**HT** **−0.6** **−0.4** **−0.7** **−0.4**	**TT** **−1.0** **−0.8** **−1.1** −0.5
Jeon et al. (2019) [54] South Korea	148 43	HT AS	PTMC	0	Transient hypoPTH: 1.4 -	14.2–53.0 m.	SF-12	General health	-				-		**-**		−1.65	

Results are displayed as differences in mean scores, except for studies by Bongers et al. (2019) [19], Lan et al. (2021) [25], Nakamura et al. (2020) [27], Nies et al. (2017) [28], and Zhang et al. (2020) [31], which are displayed as differences in median scores. Significant differences are bolded. ^a^ Postop: postoperative. ^b^ Preop: preoperative. ^c^ PTC: papillary thyroid carcinoma. ^d^ FTC: follicular thyroid carcinoma. ^e^ FVPTC: Follicular variant of papillary thyroid carcinoma. ^f^ RLNi: Recurrent laryngeal nerve injury. ^g^ Hypo-PTH: Hypoparathyroidism. **^☨^** No statistical test performed by the authors.

**Table 6:** HrQoL outcomes after thyroid surgery for qualitative and mixed-methods studies.

Study and Country	Sample Size	Surgery Extent	Pathology Details	RAI (%)	Time of QoL Assessment	Methodology	Identified Themes	Frequency of Responses (%)		Conclusion
Diamond-Rossi et al. (2020) [72] USA	47	TT	87% PTC ^a^ 13% FTC ^b^	100	0.17 to 10 y. post-RAI (mean 3.9 y. post-RAI)	Thematic analysis Focus groups	Xerostomia (dry mouth) Salivary gland dysfunction Xeropthalmia (dry eyes) Epiphora (Eye tearing) Dysgeusia (altered taste) Epistaxis Lack of knowledge and preparation for treatment Regret of treatment Distress that thyroid cancer is labeled as a “good cancer”	18.3 14.8 18.9 16.8 19.8 4.2 N/a N/a N/a		Thyroid cancer survivors reported a wide range of RAI treatment-related effects and psychosocial concerns that appear to reduce quality of life. The psychosocial concerns reported by participants underscore the significant unmet information and support needs prior to and following RAI treatment among DTC patients.
Doubleday et al. (2020) [73] USA	62	TT	DTC	N/a	Preop ^c^ Postop ^d^ 2 w. Postop 6 w. Postop 6 m. Postop 1 y.	Grounded theory analysis Semistructured interviews	Numbness and tingling Minor symptoms Interference of symptoms with life (major symptoms); Sleep disturbance: nighttime symptoms and difficult medication schedule; Unclear attribution of symptoms: muscle cramps; Symptom persistence and frustration; Concerns/problems with high calcium carbonate intake: nausea and frustration; Fear of calcium overdose; Felt self-conscious; Knew what to expect; Felt supported; Adaptation	Postop: 2 w.: 51 6 w.:27 1 y.: 40		Early postoperative transient hypoparathyroidism is common, but when appropriately managed did not have a substantial negative impact on the overall quality of life.
Hedman et al. (2017) [69] Sweden	21	TT	71% PTC 29% FTC	100	3 m.–18 y. since diagnosis (mean 4 y.)	Content analysis Semistructured interviews	Anxiety Contraindications: hidden anxiety Distrust as a source of anxiety Protective strategies	N/a N/a N/a N/a		Anxiety is a common—although partially hidden—problem in DTC survivors, as they tended to deny it early in the dialogues. As anxiety is clearly related to follow-up routines, these should therefore be reevaluated.
Kletzien et al. (2018) [70] USA	42	TT	PTC	N/a	Preop Postop 2 w Postop 6 w Postop 6 m Postop 1 y	Grounded theory analysis Semistructured interviews	Any concerns or symptoms of impaired communication	Preop: 5 Postop: 2 w.: 57 6 w.: 44 6 m.: 31 1 y.: 50		Voice changes are common after surgery for papillary thyroid cancer and affect quality of life for many patients even after 1 year of follow-up.
Nickel et al. (2019) [71] Australia	791 214	TT HT	89% PTC 11% FTC	33	2–91 w. post-diagnosis (Median 23.1 w.)	Content analysis Structured telephone interview	Overall physical symptoms Fatigue Medication issues Voice issues Throat/neck discomfort Weight concerns Autonomic symptoms Other physical symptoms Overall psychological symptoms Emotional distress Mood issues Anxiety/depression Re-evaluation of life Overall lifestyle Impact on work Increased consciousness about health/lifestyle No adverse effects or issues overall No adverse effects Minor impact relative to other issues	**HT** 54.2 28.5 15.9 9.3 8.4 7.0 5.1 10.7 19.2 14.5 3.3 2.3 1.4 4.2 1.9 2.3 34.6 34.1 0.5	**TT** 69.2 35.3 24.4 12.6 10.9 6.8 4.9 14.2 18.4 12.6 4.0 3.2 1.5 9.2 5.6 3.9 21.7 19.8 1.9	According to the results of this study, patients diagnosed with DTC report wide-ranging HrQoL issues; these seem more prevalent among patients who undergo total thyroidectomies (with or without neck dissection) rather than hemithyroidectomies. For patients with small, localized DTCs, hemithyroidectomy may offer fewer adverse effects of treatment and better HrQoL outcomes than total thyroidectomy.

^a^ PTC: papillary thyroid carcinoma. ^b^ FTC: follicular thyroid carcinoma. ^c^ Postop: postoperative. ^d^ Preop: preoperative.

## Supplementary Materials

The following supporting information can be downloaded at: https://www.mdpi.com/article/10.3390/curroncol29070350/s1, Table S1: Search Strategy.
