# Supplementary material for: Health-Related Quality of Life following Total Thyroidectomy and Lobectomy for Differentiated Thyroid Carcinoma: A Systematic Review
Source: Curr Oncol. 2022 Jun 21;29(7):4386–422. doi: 10.3390/curroncol29070350 (PMC9323259; doi:10.3390/curroncol29070350)
Supplement: Supplementary file 1 [file curroncol-29-00350-s001.zip › curroncol-1683386-supplementary.pdf]

Table S1. Search Strategy.

**Database**: Medline	**Dates:** ALL 1946 to May 19, 2021
**Keywords related to diagnosis**	exp Thyroid Neoplasms/ OR ((thyroid? or thyroidal) adj3 (neoplas* or tumo* or cancer* or malignan* or carcinom* or nodul* or adenom* or adenocarcinoma* or sarcoma* or papil* or follicular* or hurthle* or oxyphil* or medullar* or anaplast* or lymphom* or microcarcinoma* or incidentaloma*)).ti,ab,kf.
**AND**	
**Keywords related to intervention**	Thyroidectomy/ OR (Thyroidectom* or Hemithyroidectom* or Hemi-thyroidectom*).ti,ab,kf. OR ((thyroid? OR thyroidal) adj3 (surger* or surgical or lobectom*)).ti,ab,kf.
**AND**	
**Keywords related to outcome**	Quality of Life/ OR Quality-Adjusted Life Years/ OR (QOL or quality of life or life quality).ti,ab,kf.OR (HRQoL or health related quality of life or health-related quality of life or health related quality of living).ti,ab,kf.OR (quality adjusted life year* or qaly* or qald* or qale* or qtime* or life year or life years).ti,ab,kf. OR (disability adjusted life or disability-adjusted life or daly*).ti,ab,kf. OR value of life.ti,ab,kf.OR Happiness.ti,ab,kf. OR (sf36 or sf 36 or short form 36 or shortform 36 or short form36 or shortform36 or sf thirtysix or sfthirtysix or sfthirty six or sf thirty six or shortform thirtysix or shortform thirty six or short form thirtysix or short form thirty six).ti,ab,kf OR (sf6 or sf 6 or short form 6 or shortform 6 or sf six or sfsix or shortform six or short form six or shortform6 or short form6).ti,ab,kf. OR (sf8 or sf 8 or sf eight or sfeight or shortform 8 or shortform 8 or shortform8 or short form8 or shortform eight or short form eight).ti,ab,kf.OR (sf12 or sf 12 or short form 12 or shortform 12 or short form12 or shortform12 or sf twelve or sftwelve or shortform twelve or short form twelve).ti,ab,kf. OR (sf16 or sf 16 or short form 16 or shortform 16 or short form16 or shortform16 or sf sixteen or sfsixteen or shortform sixteen or short form sixteen).ti,ab,kf. OR (sf20 or sf 20 or short form 20 or shortform 20 or short form20 or shortform20 or sf twenty or sftwenty or shortform twenty or short form twenty).ti,ab,kf. OR (hql or hqol or h qol or hrqol or hr qol).ti,ab,kf. OR (quality of wellbeing or quality of well being or index of wellbeing or index of well being or qwb).ti,ab,kf.OR nottingham health profile*.ti,ab,kf. OR sickness impact profile.ti,ab,kf. OR rosser.ti,ab,kf. OR functional status questionnaire.ti,ab,kf. OR duke health profile.ti,ab,kf. OR (eq or euroqol or euro qol or eq5d or eq 5d or euroqual or euro qual).ti,ab,kf.
**AND**	
Limit to (english or french)	

**Database**: Embase	**Dates:** ALL 1974 to May 19, 2021
**Keywords related to diagnosis**	exp thyroid tumor/ OR ((thyroid? or thyroidal) adj3 (neoplas* or tumo* or cancer* or malignan* or carcinom* or nodul* or adenom*

	or adenocarcinoma* or sarcoma* or papil* or follicular* or hurthle* or oxyphil* or medullar* or anaplast* or lymphom* or microcarcinoma* or incidentaloma*)).ti,ab,kw.
**AND**	
**Keywords related to intervention**	exp thyroid surgery/ OR (Thyroidectom* or Hemithyroidectom* or Hemi-thyroidectom*).ti,ab,kw. OR (thyroid adj3 (surger* or surgical or lobectom*)).ti,ab,kw.
**AND**	
**Keywords related to outcome**	exp "quality of life"/ or exp "quality of life assessment"/ or exp "quality of life index"/ OR (QOL or quality of life or life quality).ti,ab,kw. OR (HRQoL or health related quality of life or health-related quality of life or health related quality of living).ti,ab,kw.OR (quality adjusted life year* or qaly* or qald* or qale* or qtime* or life year or life years).ti,ab,kw. OR (disability adjusted life or disability-adjusted life or daly*).ti,ab,kw.OR value of life.ti,ab,kw. OR Happiness.ti,ab,kw. OR (sf36 or sf 36 or short form 36 or shortform 36 or short form36 or shortform36 or sf thirtysix or sfthirtysix or sfthirty six or sf thirty six or shortform thirtysix or shortform thirty six or short form thirtysix or short form thirty six).ti,ab,kw.OR (sf6 or sf 6 or short form 6 or shortform 6 or sf six or sfsix or shortform six or short form six or shortform6 or short form6).ti,ab,kw. OR (sf8 or sf 8 or sf eight or sfeight or shortform 8 or shortform 8 or shortform8 or short form8 or shortform eight or short form eight).ti,ab,kw. OR (sf12 or sf 12 or short form 12 or shortform 12 or short form12 or shortform12 or sf twelve or sftwelve or shortform twelve or short form twelve).ti,ab,kw.OR (sf16 or sf 16 or short form 16 or shortform 16 or short form16 or shortform16 or sf sixteen or sfsixteen or shortform sixteen or short form sixteen).ti,ab,kw.OR (sf20 or sf 20 or short form 20 or shortform 20 or short form20 or shortform20 or sf twenty or sftwenty or shortform twenty or short form twenty).ti,ab,kw.OR (hql or hqol or h qol or hrqol or hr qol).ti,ab,kw. OR (quality of wellbeing or quality of well being or index of wellbeing or index of well being or qwb).ti,ab,kw. OR nottingham health profile*.ti,ab,kw. OR sickness impact profile.ti,ab,kw. OR rosser.ti,ab,kw. OR functional status questionnaire.ti,ab,kw. OR duke health profile.ti,ab,kw. OR (eq or euroqol or euro qol or eq5d or eq 5d or euroqual or euro qual).ti,ab,kw.
**AND**	
Limit to (english or french)	
**Database**: EBM reviews-Cochrane Central Register of Controlled Trials	**Dates:** ALL up to May 19, 2021
**Keywords related to diagnosis**	((thyroid? or thyroidal) adj3 (neoplas* or tumo* or cancer* or malignan* or carcinom* or nodul* or adenom* or adenocarcinoma* or sarcoma* or papil* or follicular* or hurthle* or oxyphil* or medullar* or anaplast* or lymphom* or microcarcinoma* or incidentaloma*)).mp.

**AND**	
**Keywords related to intervention**	(Thyroidectom* or Hemithyroidectom* or Hemi-thyroidectom*).mp.OR (thyroid adj3 (surger* or surgical or lobectom*)).mp.
**AND**	
**Keywords related to outcome**	(QOL or quality of life or life quality).mp OR (HRQoL or health related quality of life or health-related quality of life or health related quality of living).mp.OR (quality adjusted life year* or qaly* or qald* or qale* or qtime* or life year or life years).mp.OR (disability adjusted life or disability-adjusted life or daly*).mp.OR value of life.mp. OR Happiness.mp. OR (sf36 or sf 36 or short form 36 or shortform 36 or short form36 or shortform36 or sf thirtysix or sfthirtysix or sfthirty six or sf thirty six or shortform thirtysix or shortform thirty six or short form thirtysix or short form thirty six).mp. OR (sf6 or sf 6 or short form 6 or shortform 6 or sf six or sfsix or shortform six or short form six or shortform6 or short form6).mp.OR (sf8 or sf 8 or sf eight or sfeight or shortform 8 or shortform 8 or shortform8 or short form8 or shortform eight or short form eight).mp. OR (sf12 or sf 12 or short form 12 or shortform 12 or short form12 or shortform12 or sf twelve or sftwelve or shortform twelve or short form twelve).mp.OR (sf16 or sf 16 or short form 16 or shortform 16 or short form16 or shortform16 or sf sixteen or sfsixteen or shortform sixteen or short form sixteen).mp.OR(sf20 or sf 20 or short form 20 or shortform 20 or short form20 or shortform20 or sf twenty or sftwenty or shortform twenty or short form twenty).mp.OR (hql or hqol or h qol or hrqol or hr qol).mp.OR (quality of wellbeing or quality of well being or index of wellbeing or index of well being or qwb).mp.OR nottingham health profile*.mp.OR sickness impact profile.mp. OR rosser.mp.OR functional status questionnaire.mp. OR duke health profile.mp. OR (eq or euroqol or euro qol or eq5d or eq 5d or euroqual or euro qual).mp.
